# Perioperative Management of a Patient with Cold Urticaria

**DOI:** 10.3389/fmed.2017.00222

**Published:** 2017-12-18

**Authors:** Priscilla Agbenyefia, Lance A. Shilliam, Nicoleta Stoicea, Andrew Roth, Kenneth R. Moran

**Affiliations:** ^1^Department of Anesthesiology, OSU Wexner Medical Center, Columbus, OH, United States; ^2^Department of Anesthesiology, Akron General Medical Center, Akron, OH, United States

**Keywords:** cold urticaria, cold allergy, hypersensitivity reactions, immune reactions, perioperative normothermia, perioperative hypothermia, perioperative anaphylaxis, anesthesiology

## Abstract

Cold urticaria consists of an allergic immune response to cold temperatures with symptoms ranging from pruritic wheals to life-threatening angioedema, bronchospasm, or anaphylactic shock. Adequate planning to maintain normothermia perioperatively is vital due to impaired hypothalamic thermoregulation and overall depression of sympathetic outflow during deep sedation and general anesthesia. This case report describes the successful perioperative management of a 45-year-old female with a history of cold urticaria undergoing a laparoscopic Nissen fundoplication for refractory gastroesophageal reflux disease and discusses how to appropriately optimize the care of these patients.

## Introduction

Cold urticaria, a subset of chronic urticarias, was first described in the 1860s and is characterized by the presence of chronically recurring wheals for a period longer than 6 weeks after exposure to cold stimuli (1, 2). Chronic urticaria can be classified into physically induced and idiopathic types (1, 3). Physical urticarias are induced by physical stimuli such as friction, pressure, cold, or sun exposure (3). Cold urticaria comprises between 3 and 33.8% of physical urticarias (4, 5), with a higher incidence in cold climates. Within minutes of exposure to a cold stimulus, patients with cold urticaria develop a pruritic urticarial rash, which may progress to angioedema and anaphylaxis (1, 2, 6). Systemic anaphylaxis occurs in one out of three patients susceptible to cold urticarial (4). The cardiovascular system is the most commonly affected extracutaneous system, followed by the respiratory and gastrointestinal systems (4). Stimuli that can induce cold urticaria include ingestion of cold substances, handling of cold objects, exposure to cold environments, and engaging in aquatic activities (1, 2, 6).

**Figure 1:**
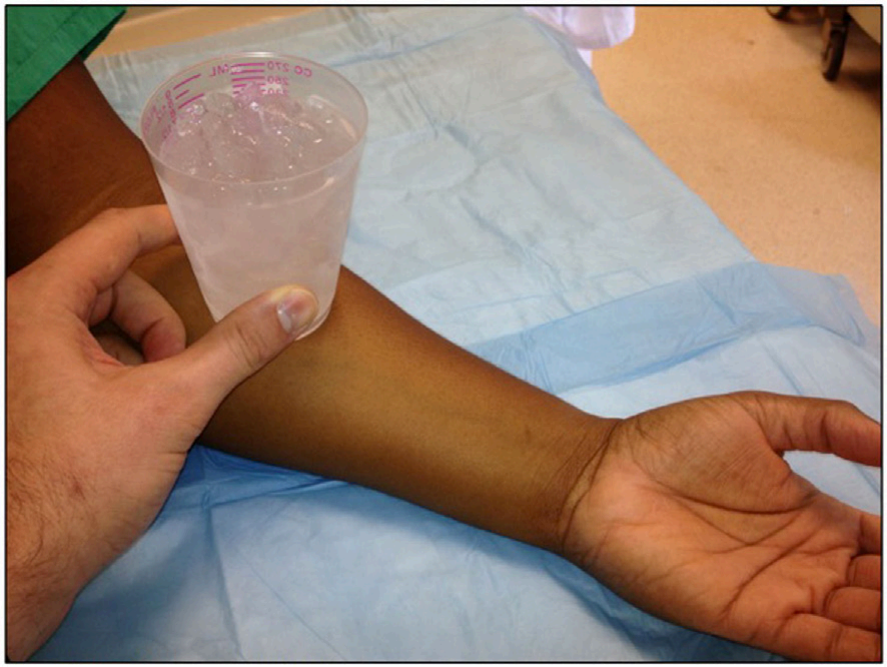
A cold stimulus (0–4°C) is placed on the forearm for 5 min. The presence of an immediate coalescent wheal is indicative of cold-induced urticaria.

Urticaria represents a type I hypersensitivity reaction. Through an unclear mechanism, cold stimuli lead to mast cell or basophil degranulation, followed by the release of histamine and other inflammatory mediators (3, 7). The estimated incidence of cold urticaria is 0.05% generally, with young adults most frequently affected, and women twice as likely to be affected as men (5). The mean duration of the disorder is 4.8–9.3 years, but it can last up to 20 years (1). Diagnosis can be confirmed by placing a cold stimulus (0–4°C) on the forearm for 5 min (2) (as shown in Figure 1). The presence of an immediate coalescent wheal is indicative of cold-induced urticaria (2). Immersion of a hand in 10°C water for 5 min may also be used, but there may be an increased risk of angioedema or systemic reactions with this method (1).

Cold urticarias are rarely associated with underlying disease. However, some infectious diseases, medications, and other pathologies [notably cryoglobulinemia and cryopyrin-associated periodic syndrome (CAPS)] have been associated with cold urticaria (8). In cryoglobulinemia, patients have increased immunoglobulin levels in the serum, which predisposes them to reversible precipitation under lower temperatures. CAPS is a rare genetic disorder caused by mutations in inflammatory pathways leading to increased IL-1β production. It is characterized by cold urticaria, arthralgias, fevers, renal amyloidosis, sensorineural hearing loss, conjunctivitis, chronic aseptic meningitis, and mental retardation (8). Infectious diseases associated with cold urticaria include syphilis, varicella, hepatitis, and some respiratory viral infections (1). Penicillin, griseofulvin, and angiotensin converting enzyme inhibitors have been implicated as well (1).

## Case Report

A 45-year-old white female with a long-standing history of uncontrolled gastroesophageal reflux disease presented for laparoscopic Nissen fundoplication. The patient had a 14-year history of cold urticaria in addition to osteoarthritis, diverticulitis, and restless leg syndrome. Prior triggers for the patient’s cold urticaria included consuming ice chips, being in cold environments and colonoscopy bowel preparation. She was also allergic to naproxen, guaifenesin, and menthol. Her previous cold exposures had led to complications as varied as extremity numbness to airway compromise, anaphylaxis, and cardiac arrest.

Her home medications included escitalopram, pramipexole, cetirizine, and ranitidine. Her surgical history consisted of a cesarean delivery and cholecystectomy, both prior to the diagnosis of cold urticaria. Neither she nor her family had a history of adverse reactions to anesthesia. At presentation for surgery, the patient’s physical exam was unremarkable, including a Mallampati class II oropharynx. Vital signs before induction were normal: heart rate 82 beats/min, blood pressure 122/80 mmHg, respiratory rate 16/min, SpO_2_ 100% on room air.

Thirty minutes prior to induction, diphenhydramine 25 mg IV, famotidine 20 mg IV, hydrocortisone 100 mg IV, and midazolam 2 mg IV were administered. Intubation was uneventful after induction of general anesthesia with lidocaine, propofol, and fentanyl. Rocuronium was used for neuromuscular blockade, and anesthesia was maintained with desflurane. Normothermia was maintained with two warm blankets pre-intubation upon arriving to the OR and 2 separate forced air warming blankets over the lower and upper body after intubation. The OR temperature was maintained above 70°F. A fluid warmer was used on all intravenous fluids flowing into an 18-G peripheral venous catheter. Body temperature was monitored with both an oropharyngeal probe and a bladder probe. Epinephrine was prepared in advance and kept immediately on hand. Just before incision, the patient developed hypotension with a blood pressure of 69/30 mmHg, which was successfully treated with epinephrine. There was no evidence of urticarial rash or bronchospasm during this period. Hypertension and tachycardia resulting from epinephrine administration were treated with esmolol and by increasing the depth of anesthesia. There were no more episodes of hemodynamic instability for the remainder of the case.

The surgical procedure progressed without complication. Paralysis was reversed with neostigmine, and extubation was uneventful. Hydromorphone was used for pain control during and after the case. In the postoperative care unit, the patient initially showed delirious disorientation to time and place, but this resolved after haloperidol 2 mg IV. Her vital signs and temperature remained stable during the recovery period with no evidence of an urticarial rash. Warm blankets were utilized to maintain normothermia while recovering from general anesthesia.

## Discussion

Patients with cold urticaria undergoing general anesthesia risk developing anaphylaxis unless strict perioperative normothermia is maintained. Redistribution of core body temperature peripherally occurs within the first 30 min to an hour following induction of general or regional anesthesia as a result of sympathoplegia and vasodilation (9). This can lead to a 1–2°C drop in core temperature if no rewarming efforts are implemented to eliminate or reduce the core-peripheral body temperature gradient. Further decreases in temperature ensue due to impaired hypothalamic thermoregulation by anesthetic agents and reduced threshold for shivering (9). In this case, normothermia was achieved with forced air warming blankets, intravenous fluids warmed with an in-line fluid warmer to 40°C, and careful adjustments to ambient room temperature. The objective was to maintain normothermia at all times.

The literature concerning perioperative management of cold urticarial is scant. In the majority of reported cases, premedication included antihistamine agents (both H_1_ and H_2_ receptor blockers) and a single dose of corticosteroids. When providing general anesthesia for a patient with cold urticaria, prevention of the initial as well as final mechanism of anaphylaxis is a primary goal. To that end, famotidine, diphenhydramine, and a single dose of hydrocortisone administered intravenously 30 min prior to induction may deter immune histamine release. In addition, medications known to induce histamine release should be avoided as much as possible. While histamine release from drugs is not known to trigger cold urticaria, an urticarial reaction from any source can confuse the clinical picture of a patient with cold urticaria. Neuromuscular blocking agents such as rocuronium have been implicated in up to 58.2% of anesthetic-associated anaphylaxis (10). In a study that estimated the rate of anaphylaxis during anesthesia, rocuronium was associated with 56% of cases, succinylcholine with 21%, and vecuronium with 11% (10). Nonetheless, in this case, rocuronium was chosen over other paralytics because of its rapid onset and ease of reversal. In retrospect, the use of a non-depolarizing paralytic other than rocuronium may be preferable.

Morphine, codeine, and some synthetic opioids are known to increase histamine levels independent of IgE antibodies (11). Anesthetics such as propofol and thiopental are rare causes of anaphylaxis as well (11, 12). Other agents used in the operating room that may provoke an immune response include midazolam, ketamine, chlorhexidine, colloids, protamine sulfate, and local anesthetics such as lidocaine (11). While completely avoiding the risk of a histamine or immune-mediated reaction is not possible, minimizing the risk may prevent confusion that could result from a non-cold related reaction.

The patient’s hypotensive episode prior to incision was not associated with cutaneous symptoms, bronchospasm, or hypothermia. Since hypotension after induction of general anesthesia is not uncommon, and several drugs used during anesthesia can induce hypotension, the reaction did not definitively point to cold urticaria. However, although hypothermia triggers cold urticaria, systemic hypothermia is not required to make a diagnosis as a cold stimulus can elicit an immune response without a significant drop in core temperature. For instance, in delayed-type cold-induced urticaria, symptoms develop 24–72 h after exposure, even after rewarming (2). Reactions precipitated by consuming and handling cold substances such as ice chips, which are triggers for this patient, are seldom associated with hypothermia. In addition, premedicating with diphenhydramine, famotidine, and hydrocortisone may have blunted the immune response, resulting in an unremarkable physical exam.

The patient’s development of delirium after emerging from anesthesia could be ascribed to the side effects of anesthesia, combined with side effects of preoperative famotidine, diphenhydramine, and hydrocortisone (13). Either of these factors separately, or a multifactorial etiologies could have caused delirium in this patient. Other elements could have played a role as well.

Second-generation H_1_ antihistamines at standard doses are first line therapy for treating and preventing chronic urticarias. Many first-generation antihistamines have shown similar efficacy, but their sedating, antimuscarinic and anti-alpha-adrenergic properties make them unfavorable for outpatient management (14). Because it is difficult to control chronic urticarias, especially in patients who do not respond to first line therapy, there is no specific protocol for treatment. Some studies have used adjunctive remedies to H_1_ blockers with success. Doses of H_1_ antihistamines up to four times the usual dose have increased prophylactic and abortive treatment response from 45% to more than 60% (14). Results from studies evaluating the combination of H_1_ and H_2_ receptor antagonists are inconsistent, although the combination is still preferred to other second line treatment options. Limited use of corticosteroids as a second line agent has yielded promising results, but is only recommended as short-term abortive and prophylactic therapy due to the side effects of chronic use (14).

Leukotriene receptor antagonists, anti-inflammatory agents (dapsone, sulfasalazine, hydroxychloroquine, colchicine), immunosuppressant agents (cyclosporine, methotrexate, cyclophosphamide, azathioprine, sirolimus, mycophenolate mofetil) and biological agents such as intravenous immunoglobulin and antibodies to IgE (Omalizumab) have all yielded promising results in the treatment of patients with antihistamine-resistant chronic urticaria (14–16). For patients with cold urticarias due to CAPS, various agents targeting interleukin-1 and interleukin-1β have shown favorable results (8). All patients with cold urticaria should be provided with epinephrine autoinjectors for emergency situations.

## Conclusion

Cold urticaria is a rare and potentially deadly disease that poses an easily underestimated challenge for anesthesiologists. Proper preoperative management is critical to prevent catastrophic events, such as angioedema or anaphylaxis. The risk of severe reaction can be mitigated through careful planning for seamless perioperative normothermia, and preoperative prophylactic use of histamine 1 and histamine 2 receptor blockers and corticosteroids. Normothermia is best achieved with a single or multiple forced air warming blankets and careful monitoring of core and ambient temperature in the operating room. Avoidance of potentially allergenic medications may simplify recognition of the true cause of any reaction. These aggressive steps will minimize the perioperative morbidity and mortality in patients with cold urticaria.

## Ethics Statement

We have written informed consent to use the patient’s information for publication of this paper.

## Author Contributions

PA, first author: drafting, acquisition, and interpretation of data. NS, AR, and LS: revision of content. KM, corresponding author: drafting, acquisition, analyzing, and interpretation of data.

## Conflict of Interest Statement

The authors declare that the research was conducted in the absence of any commercial or financial relationships that could be construed as a potential conflict of interest.
